# Investigation of a Magnetic Sensor Based on the Magnetic Hysteresis Loop and Anisotropic Magnetoresistance of CoFe Thin Films Epitaxial Grown on Flexible Mica and Rigid MgO Substrates with Strain Effect

**DOI:** 10.3390/mi16040412

**Published:** 2025-03-30

**Authors:** Jen-Chieh Cheng, Min-Chang You, Aswin kumar Anbalagan, Guang-Yang Su, Kai-Wei Chuang, Chao-Yao Yang, Chih-Hao Lee

**Affiliations:** 1 Institute of Nuclear Engineering and Science, National Tsing Hua University, Hsinchu 30013, Taiwan; jeff89042@gmail.com (J.-C.C.); jk733270107@gmail.com (K.-W.C.); 2 Department of Engineering and System Science, National Tsing Hua University, Hsinchu 30013, Taiwan; ghkl22025@gmail.com (M.-C.Y.); aswinakumar89@gmail.com (A.k.A.); watch159159159@gmail.com (G.-Y.S.); 3 Department of Materials Science and Engineering, National Yang Ming Chiao Tung University, Hsinchu 30010, Taiwan; cyyang8611@nycu.edu.tw

**Keywords:** mica, CoFe films, MgO, AMR magnetic sensor, flexible sensor, epitaxial growth

## Abstract

The anisotropic magnetoresistance (AMR) effect is widely used in microscale and nanoscale magnetic sensors. In this study, we investigate the correlation between AMR and the crystal structure, epitaxial relationship, and magnetic properties of Co_50_Fe_50_ thin films deposited on rigid MgO and flexible mica substrates. The AMR ratio is approximately 1.6% for CoFe films on mica, lower than the 2.5% observed in epitaxially grown films on MgO substrates. The difference is likely due to the well-defined easy axis in the single domain epitaxial thin films on MgO, which enhances the AMR ratio. Microscopic strain induced by lattice mismatch and bending on flexible substrates were determined using grazing incidence X-ray diffraction and extended X-ray absorption fine structure techniques. These results showed that neither microscopic nor macroscopic strain (below 0.5%) affects the AMR ratio on mica, suggesting its suitability for magnetic sensors in flexible and wearable devices. Additionally, investigating M-H loops under various growth temperatures, lattice mismatch conditions, and bending strains could further benefit the fabrication and integration of the micro-scale magnetic sensors in the microelectronic industry.

## 1. Introduction

Anisotropic magnetoresistance (AMR) devices use the resistance change when the current direction is different from the applied magnetic field as a magnetic sensor. The resistance as a function of included angles between applied current direction and sensing magnetic field is measured [1]. As a commercial micro-to-nano scale magnetic sensor in the microelectronic industry, applying the principle of magnetoresistance is the easy way of reducing the size. In addition, during the device fabrication, the preparation of thin film and packaging, the thermal stress, and the mismatch of heterojunction materials cause inevitable strain and stress, which usually affects the performance of magnetic sensors profoundly. The strain problem is especially important when the wearable device is to be implemented.

In reality, AMR devices exhibit lower sensitivity compared to their counterpart, giant magnetoresistance (GMR) and tunnel magnetoresistance (TMR) sensors. However, an AMR device is much easier to fabricate than GMR and magnetic tunnel junction structures. The simple structure of AMR is advantageous for industrial applications. For example, the wide range of AMR applications can be found in [1]. AMR sensors can be used in magnetic devices such as hard drive read heads [2], traffic speed detection systems, and wearable detection devices [3]. They can also serve as health monitoring devices when applied to flexible substrates. Many studies and commercial products, such as magnetic detection systems, electromagnetic field detectors, switches [4], and e-skins [5,6,7], have been reported for these small sensors. The ability to convert magnetic movement signals under an external magnetic field into electric signals is the main advantage of this type of magnetic sensor. In addition, the GMR and TMR cannot be used in a high dynamic range of a magnetic field when both the ferromagnetic layers are magnetized in the same directions as the lower field. The single ferromagnetic layer of the AMR device can be applied to a much larger dynamic range of magnetic field.

Today, a high AMR ratio is observed in ferromagnetic materials such as Fe, Co, and Ni. It has been observed that a higher AMR ratio can be achieved by combining these three ferromagnetic materials, for example, in NiCo, NiFe, CoFe, and FeCoNi. NiFe is the most widely used AMR material in industrial applications, exhibiting an AMR range from 0.2 to roughly 5% at room temperature [8,9,10]. To explore other potential materials that could also enhance a higher AMR, CoFe is one of the most likely candidates. CoFe is a well-known material in most magnetic works due to its high thermal stability and magnetization. For instance, CoFe has been extensively studied in the spintronics industry, such as magnetic random access memory [11]. While NiFe AMR is well studied, there is scarce data available on the AMR properties of CoFe [12,13]. Whether CoFe could be a potential AMR material still needs to be studied. On the other hand, the ferromagnetic materials are not the only important parts of the AMR devices; the substrates are also important for the deposition materials. A proper substrate could also affect the device’s performance. For thin-film AMR sensors, the magnetic property may not just affect the magnetic field, the growth of the thin film is also important. The AMR sensors in the industry are mostly polycrystal with rigid substrate. Recently, wearable devices have gained more attention in the industry. For such kinds of devices, a flexible substrate is needed. Meanwhile, the strain may play a role in the process of applied strain. When a flexible magnetic device is under an applied magnetic field, the magnetostriction effect becomes important. Currently, the CoFe film has been extensively studied in many works on magnetostriction. It has been found to exhibit an extremely high magnetostriction coefficient, exceeding 1000 ppm [14], in Co_50_Fe_50_. In Hunter’s findings, magnetostriction was also correlated with the phase diagram, particularly when the concentration approaches the boundary of the mixed (fcc + bcc)/bcc phase in CoFe. The phase diagram, calculated based on theoretical predictions, is shown in [15,16]. This is one of the reasons we use Co_50_Fe_50_ (CoFe) in this work.

On the other hand, it has been reported that the theoretical calculation of a higher AMR ratio could be seen under the same materials with better crystallinity. The review work of Ritzinger and Výborný [1] showed that crystal symmetry is one extra term when discussing the crystalline AMR ratio. Compared with the non-crystalline (or polycrystal) [1,17] case of AMR ratio, the higher AMR ratio is expected to be observed in better crystalline systems. The main difference is that non-crystalline AMR primarily considers the electric current and external magnetic field, while higher-order crystalline or epitaxy contribute additional symmetry effects, resulting in a relatively higher AMR. For this reason, it is meaningful to understand whether crystallinity affects the AMR ratio. During the fabrication of a rigid substrate and a flexible substrate, the lattice mismatch is an important parameter used to understand the difference. Thus, proper flexible and rigid substrates with compatible lattice mismatch are introduced.

To identify suitable flexible substrates, several approaches can be explored. One potential method involves depositing magnetic films on piezoelectric substrates and applying an electric field [18,19]. Another strategy focuses on growing magnetic films on flexible substrates, utilizing mechanical force through the Villari effect [20]. When subjected to an external magnetic field, materials with a positive magnetostrictive constant elongate along the magnetic field direction. This phenomenon may be one of the advantages of the development of AMR devices. However, most flexible devices constructed on polymer-based substrates cannot withstand high temperatures, and maintaining stability under high temperatures remains a challenge for such flexible devices. Therefore, there is an urgent need for alternative flexible substrates with higher melting points and performance comparable to rigid substrates.

Mica (muscovite) has been studied in many research areas, such as straintronic and wearable devices [3,21]. Mica has advantages, including thermal stability, biocompatibility, mechanical flexibility, high surface energy, and controllable cleavage [22,23,24]. The weak van der Waals forces enable thin films to grow epitaxially on mica substrates. These physical properties have the strength to develop flexible AMR magnetic sensors [3,25,26]. Therefore, mica is the flexible substrate we studied here.

Although a flexible substrate has many advantages, its main drawback is a relatively low AMR ratio. It is also important to understand the AMR ratio of the rigid substrate. Thus, a comparison between flexible and rigid substrates is essential. The rigid substrate must obtain a relatively lower lattice mismatch and higher thermal stability than mica. Thus, the MgO substrate is a suitable choice due to its lower lattice mismatch with CoFe compared to CoFe deposited on silicon or other common rigid substrates. As mentioned earlier, the flexible mica and rigid MgO are suitable substrates for comparing the AMR ratio and other related magnetic and crystal properties. Recently, some works have reported on similar topics. For example, some research groups have studied the AMR effect on epitaxial films deposited on rigid substrates [27,28,29,30,31], and similar results on single-crystal films have also been investigated [13,32,33]. While most of the AMR ratio is positive, negative AMR values have also been reported [27,29]. The phenomenon could be explained by the possibility of observing the inverse curve of the AMR ratio. To further understand the changes in AMR and other magnetic properties, research on lattice mismatch [30], thermal treatment [34,35,36], and magnetic fields higher or lower than the anisotropy field [37] has been conducted.

On the other hand, thermal stability is also important for crystal growth or related magnetic properties. Therefore, it is intriguing to examine measurements conducted at growth temperatures (T_g_) of 25, 300, 400, and 500 °C and to compare the AMR and related H_c_ and M_s_ on both the rigid MgO and flexible mica substrates. For a real magnetic sensor device, the M-H loop is important. For example, H_c_ defines the application dynamic range of the magnetic field. Meanwhile, the H_c_ could be affected by the dislocations and changes in the coherence length when the strain is applied. The higher squareness gives the largest remnant magnetization value still work in a sensor. A high squareness gives the magnetic sensor an abrupt change of turning on and off.

In this work, the highest AMR ratio of CoFe/mica is 1.6%, which is smaller than that of CoFe/MgO (2.5%), although both CoFe thin films on mica and MgO exhibit epitaxy. However, the quality of the epitaxial growth of CoFe on mica and MgO differs significantly. The higher AMR ratio of CoFe/MgO could be attributed to a well-defined easy axis and a lower lattice mismatch compared to CoFe/mica. Furthermore, the strain analysis of CoFe/mica indicates that the AMR ratio is not significantly affected by either microscopic or macroscopic strain, suggesting that CoFe on flexible mica could be a promising candidate for magnetic sensor applications on wearable devices.

## 2. Materials and Methods

CoFe thin films were prepared by DC magnetron sputtering, using a 2-inch, 99.99% pure Co_50_Fe_50_ sputter target. The films were deposited to a thickness of approximately 50 nm on flexible mica and rigid MgO substrates, at T_g_ values of 25, 300, 400, and 500 °C, to study the effects of growth temperature during the deposition process. The crystallinity and epitaxial relation as functions of T_g_ were analyzed for both CoFe/mica and CoFe/MgO samples using synchrotron-based X-ray diffraction (XRD) and phi scan measurements conducted at beamline 17B of the National Synchrotron Radiation Research Center (NSRRC) in Taiwan. The 8-circle diffractometer was used for epitaxial and in-plane XRD measurements. The symmetry of epitaxial orientation between substrates and epitaxial thin films was determined with the azimuthal scan (phi scan) about the axis perpendicular to the plane-normal. Grazing incidence X-ray diffraction (GIXRD) was also performed to determine the in-plane strains of the epitaxial films. The Polarized X-ray absorption spectroscopy (XAS) was performed at beamline 17C. The Fe K-edge (7.112 keV) and Co K-edge (7.709 keV) were calibrated using standard Fe and Co foils, with appropriate filters to minimize unwanted elastic scattering signals. Extended X-ray absorption fine structure (EXAFS) data were collected using a Lytle detector at energies 50~1000 eV above Fe and Co absorption K-edges [38]. The samples were measured in two different directions, in-plane and plane-normal, to compare strains along these directions. After data collection, EXAFS analysis was performed using the Artemis program [39]. The X-ray absorption data were converted into the bond distances around specific elements via Fourier transform. EXAFS analysis is essential for a very disordered material with very low diffraction intensity, which is not able to be measured by XRD. EXAFS is also applied to determine the bond lengths of a thin film when the weak diffraction peaks overlap with the strong substrate peaks. In our CoFe thin film on mica, some of the plane-normal CoFe peaks were obscured by the strong diffraction mica peaks. The EXAFS was used to determine the lattice spacings.

Magnetization measurements were conducted using vibrating sample magnetometry (VSM) with a Lake Shore Cryotronics PMC-3900 instrument (Lake Shore Cryotronics, Inc., Westerville, OH, USA). To investigate the strain-induced magnetization of CoFe thin films, mica substrates were exfoliated to a thickness of 50 μm. The sample was placed on 3D-printed bending molds made of polyethylene designed to apply strain. Surface morphology changes of the CoFe thin films were analyzed using a scanning electron microscope (SEM; JEOL JSM-7610F, JEOL Ltd.,Tokyo, Japan) at room temperature.

**Figure 1:**
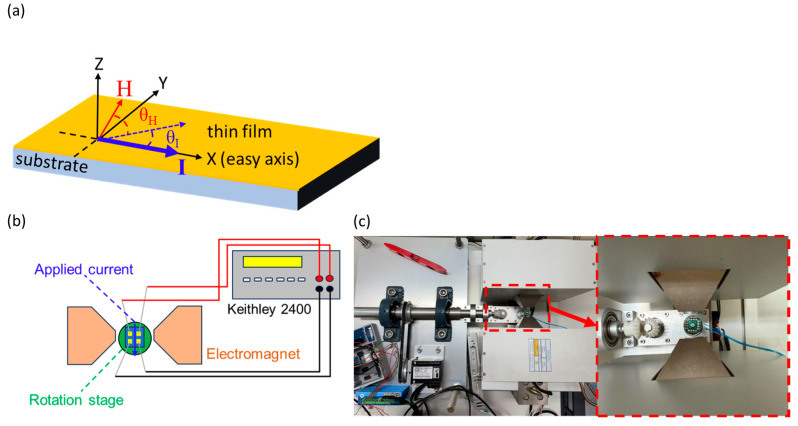
(**a**) Geometry of the in-plane AMR measurement, the θ_I_ defined as the in-plane direction of the CoFe (100), the θ_H_ defined as the angle between the applied magnetic field and the applied current, and the angle from 0° to 180° due to the uniaxial characteristic of AMR. (**b**) Schematic diagram of AMR measurement setup. (**c**) The top view of the experimental setup of the AMR measurement, with an enlarged view marked by a red line shown in the left panel.

The geometry of the in-plane AMR measurement is shown in Figure 1a. Figure 1b,c illustrates the experimental setup. When measuring the AMR ratio, the sample was placed at a rotation stage, which rotates the sample azimuthally about the plane-normal. For AMR measurements, electrodes were prepared by depositing a 100 nm-thick Au layer on top of the CoFe thin films via DC sputtering. The AMR measurements were conducted with a 100 μA current applied along two crystal axes of the CoFe film, which exhibit four-fold symmetry. The only difference is that the CoFe on mica has 3 different domains. The current angle on AMR measurement is as follows: θ_I_ = 0° was defined as CoFe (100), corresponding to the *X*-axis in Figure 1a, and θ_I_ = 45° is along the CoFe (110). The θ_H_ was defined as the included angle between the magnetic field (2500 Oe in this study) and the applied current. The θ_I_ and θ_H_ were determined relative to the easy axis and applied current direction. The in-plane easy axis can be obtained by measuring the highest squareness of the M-H loop among the data at different angles. The in-plane crystallographic orientation can be observed by the grazing incidence XRD experiments. The AMR measurement considered only in-plane directional changes. The AMR response was assessed using a Keithley 2400 source meter with a four-probe method.

## 3. Results and Discussions

### 3.1. Epitaxial Relationship with Change of AMR Ratio

The epitaxial relationship between CoFe and the single crystal substrate was determined by a four-circle diffractometer in standard Eulerian geometry. A four-circle diffractometer consists of four independent rotation axes: Two theta, Theta, Chi, and Phi circles. The sample plane-normal was aligned with the rotation axis of the phi circle. These four circles are: Two Theta: rotation of the detector arm controlling the X-ray scattering angle; Theta: rotation of the sample stage around an axis perpendicular to the incident X-ray beam; Chi: tilting of the sample axis, controlling the inclination of the crystal’s normal with respect to the incident beam; Phi: rotation of the sample around its own plane-normal azimuthally, allowing different crystal planes to be measured.

**Figure 2:**
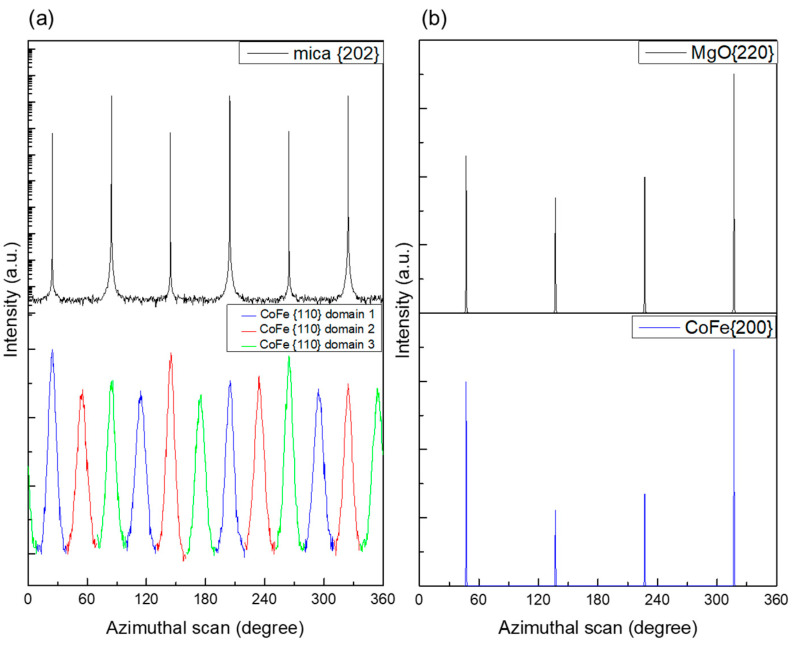
Azimuthal scan of the (**a**) mica{202} (left-top) and CoFe{110} (left-bottom) plane. The epitaxial data on mica were taken from our previous work [40], and the (**b**) MgO{220} (right-top) and CoFe{200} (right-bottom) plane is also shown for CoFe films grown on the MgO (001) substrate at 500 °C.

The result of the epitaxial growth of CoFe/mica and CoFe/MgO is illustrated in Figure 2. The azimuthal scan results clearly demonstrate that the crystallographic orientation of CoFe on MgO is CoFe (001)[100]//MgO(001)[110] in a single domain, while, on mica, it is in CoFe (001)[100]//mica(001)[100] with three equal domains. In Figure 2a, the detailed azimuthal scan is as follows: mica{202} and CoFe{101} azimuthal scan is rotated about the CoFe (001) or mica (001) axis with a fixed included angle of Chi = 45° (for CoFe) and 35.3° (for mica) between the rotation axis and mica{020} circular scan. The azimuthal scan on mica confirms the epitaxial relationship between CoFe{101} and mica{202}. The three domains, each rotated by 120°, manifest a four-fold symmetry within each domain. Since CoFe{101} and CoFe{011} are perpendicular to each other, each diffraction peak appears at an azimuthal angle of 30°, resulting in twelve diffraction peaks [40]. Based on the body-centered cubic structure of CoFe, the preferred orientation of CoFe films in the plane-normal direction is {002}, as indicated by the location of the diffraction peaks of CoFe{101} and CoFe{011} at Chi = 0°. CoFe{100} aligns with the a-axis of the mica unit cell. For the epitaxial CoFe grown on MgO (001), Figure 2b illustrates the epitaxial relation between CoFe films {200} and MgO substrate {220}, displaying a four-fold symmetry.

**Figure 5:**
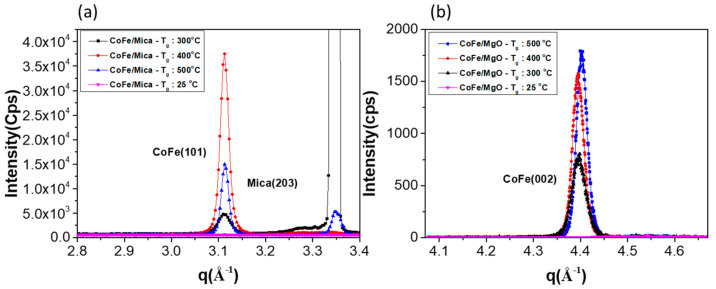
The plane-normal XRD result as a function of growth temperature in (**a**) CoFe (101) on mica substrate and (**b**) CoFe (002) on MgO substrate. The calculated coherence lengths are listed in Table 2. The q is defined as 4πsinθ/λ (λ = 1.5498Å).

Table 1 presents a comparison of the lattice d-spacing of the CoFe (100) plane from the ICSD database [41,42] and the experimental data obtained from our plane-normal XRD measurements shown in Figure 5. For CoFe/mica, the lattice constants along the a-axis for mica and bulk CoFe are 5.304 Å and 2.855 Å, respectively. To calculate the lattice mismatch, the a-axis lattice constant of CoFe on mica is taken as twice that of the unit cell, resulting in a value of 5.710 Å to match the lattice constant of mica (100) on the interface. For CoFe/MgO, the d-spacing is 2.978 Å for bulk MgO (110) and 2.855 Å for bulk CoFe (100). The XRD results indicate lattice constants along the a-axis of the 5.308 Å for mica and 5.710 Å for CoFe in the CoFe/mica system. Similarly, for the CoFe/MgO system, the d-spacings are 2.976 Å for MgO (110) and 2.874 Å for CoFe (100). The presence of negative or positive lattice mismatch can induce compressive or tensile strain in the thin film, which may also contribute to the higher or lower AMR ratio observed on MgO and mica substrates. The correlation between lattice mismatch and the related AMR ratio is discussed in a later section.

**Figure 3:**
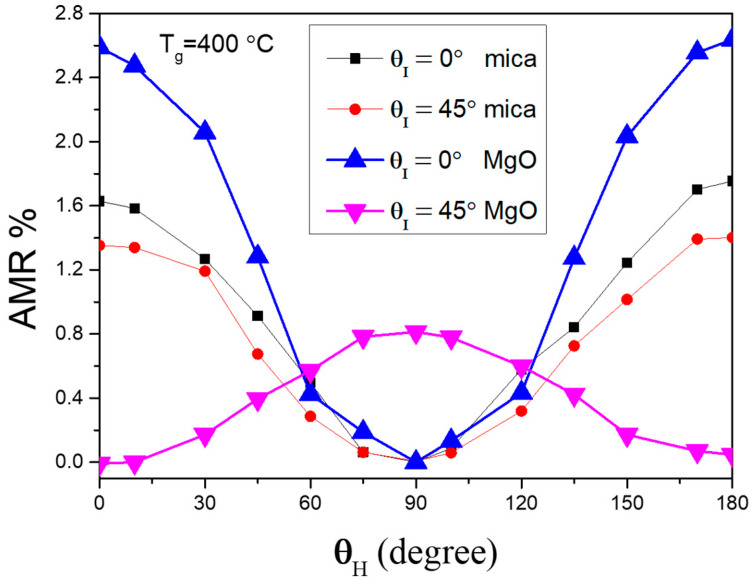
AMR ratio of CoFe films on mica substrate and MgO substrate. θ_H_ is defined as the included angle between the applied magnetic field and applied current directions, and θ_I_ is defined as the included angle between CoFe [100] and the applied current directions. The AMR data on mica were taken from our previous work [40].

The highest AMR ratio for films grown at T_g_ = 400 °C on a mica substrate is about 1.6%, as shown in Figure 3. However, the AMR ratio is lower than that of MgO, which is about 2.5% at θ_I_ = 0^°^. For the AMR ratio grown on MgO, our results are consistent with the work of Zeng et al. [13], which has the same epitaxial relationship. However, this is the case only for the MgO substrate. When other polymer substrates are considered, mica becomes a better choice. Our previous study on CoFe/mica showed that a higher AMR ratio is achievable with improved epitaxy compared to other polymer substrates [40]. CoFe thin film grown on polymer substrates are polycrystalline with no epitaxial structure. The non-epitaxial CoFe thin film on an amorphous polymer substrate exhibits a lower AMR ratio. Mica, however, is a unique case; it exhibits epitaxy but with three domains rather than forming a fully powdered CoFe thin film. This partially epitaxial structure on mica results in a higher AMR ratio compared to powdered CoFe on polymer substrates, indicating that mica has the highest crystallinity among polymer substrates. These results suggest that improved epitaxial quality leads to a higher AMR ratio.

The AMR ratios, as functions of θ_H_, differ significantly between CoFe grown on mica and MgO. One of the differences in the CoFe/MgO system is its lower AMR ratio at different θ_I_ values. Specifically, the lowest AMR ratio occurs at θ_I_ = 45°, which is a different behavior from the CoFe/mica case. The distinct trends in AMR ratios for θ_I_ = 0^°^ and 45^°^ on MgO could be due to the spin–orbital interactions, as suggested by band structure calculations in Zeng’s work [13]. Additionally, the θ_H_ dependence is completely different between CoFe on MgO and mica samples. This discrepancy could be due to the fact that the epitaxial CoFe film on MgO is a single domain, whereas CoFe on mica forms three symmetric domains, which may smear out in-plane anisotropy and reduce any significant preferred orientation. Another possible explanation is the angle between θ_I_ and the easy axis of CoFe. Previous studies by Miao et al. [27] and Yang et al. [37] have reported that AMR curves could be in different phases due to the different angles between θ_H_, θ_I_, and the easy axis. Their results show that angle-dependent AMR measurements could result in opposite AMR ratio values when θ_I_ is aligned along or away from the easy axis. This could explain the observed phase differences in the AMR curves shown in Figure 3.

An interesting finding reported by Du et al. [43] suggested that the easy axis could align in the same direction as the maximum change in the AMR ratio. In the case of CoFe/MgO, the higher AMR ratio along θ_I_ = 0° is very close to its easy axis, whereas θ_I_ = 45° is away from the easy axis, which could result in a different AMR curve. Additionally, the compressive or tensile stresses applied to CoFe due to the lattice mismatch (see Table 1) might also play a role. Furthermore, the magnetic properties might provide more information for fabricating a magnetic sensor and are discussed in the following section.

### 3.2. The M-H Curve, Coherence Length, and Particle Size as Functions of Growth Temperature

**Figure 4:**
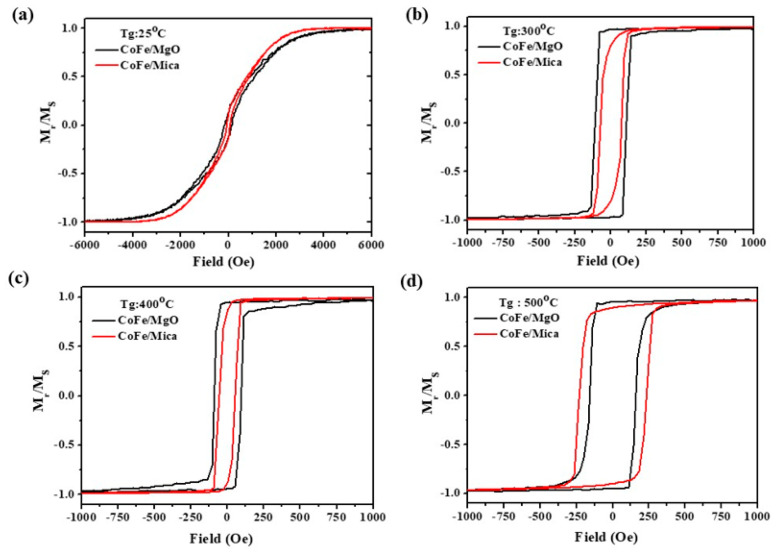
Hysteresis loops of CoFe/mica and CoFe/MgO films grown at (**a**) 25 °C, (**b**) 300 °C, (**c**) 400 °C, and (**d**) 500 °C, taken in an in-plane field reversal.

Understanding the changes in magnetic properties, such as H_c_ and squareness, is important when preparing a good magnetic sensor. The M-H curve reveals variations in H_c_ and squareness, which are significantly influenced by T_g_. In Figure 4a–d, H_c_ increases with higher T_g_ for both MgO and mica substrates until T_g_ = 400 °C. The H_c_ of CoFe/MgO is larger than that of CoFe/mica. In the meantime, larger squareness in CoFe/MgO might be attributed to a better epitaxial single domain, with the in-plane easy axis along the applied magnetic field direction.

However, when T_g_ = 500 °C, the H_c_ becomes larger on mica rather than on MgO, as shown in Figure 4d. A similar phenomenon was previously investigated by Littlejohn et al. [44]. Growth at higher temperatures results in the desorption of potassium, thereby affecting and degrading the structure of mica, thus reducing the crystallinity of CoFe. While the mica substrate at T_g_ = 500 °C may face degradation issues, signs of resilience against decomposition from the mica substrate were observed. Consequently, at 500 °C, CoFe films on both MgO and mica substrates exhibit a rapid increase in H_c_, aligning well with larger CoFe particle sizes.

**Figure 6:**
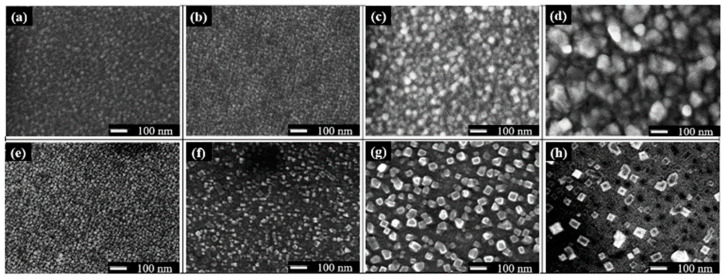
SEM images of CoFe/mica films grown at (**a**) 25 °C, (**b**) 300 °C, (**c**) 400 °C, and (**d**) 500 °C, and CoFe/MgO films grown at (**e**) 25 °C, (**f**) 300 °C, (**g**) 400 °C, and (**h**) 500 °C. The details of the CoFe particle size are shown in Table 2.

During the measurement of the M-H loop, it was found that the M-H loop could be affected by the particle size and coherence length of the CoFe thin film. Experimental data were obtained from SEM and XRD to understand the particle size and coherence length of the CoFe film on both substrates that affect the M-H loop. Figure 6a–h shows the particle size of CoFe under different T_g_ values, and the particle sizes for both substrates are summarized in Table 2.

From the SEM results in Figure 6e–h, it can be observed that the particle shape of CoFe/MgO is different compared to CoFe/mica. This change of particle size in surface morphology could result from differences in surface energy. The surface energy of mica is approximately 0.12 J/m^2^ [45], whereas that of MgO typically falls somewhere around 1.5 J/m^2^ [42]. The surface energies of Co and Fe are about 2.5 J/m^2^ [46]. Due to the larger energy difference between CoFe films and both substrates, CoFe tends to form islands to reduce the contact area at the beginning of thin film growth. The particles on the surface of CoFe on MgO at 300 °C, 400 °C, and 500 °C resemble a square shape due to the four-fold symmetry of MgO substrates. In this part, the particle size was determined from the morphological observations of real-space SEM images, while the coherence length was calculated from the XRD results in the reciprocal space using the Debye–Scherrer equation [47], as shown in Equation (1) as follows:

(1) $$D=\frac{K\lambda }{\beta cos\theta }$$

where “D” represents the crystalline size of the nanoparticles, “K” stands for the Scherrer constant, “λ” denotes the wavelength, and “β” represents the full width half maximum (FWHM) of the diffraction peak.

For the change in particle size, CoFe films at T_g_ = 25 °C exhibited no noticeable particle formation. The particle size increased as T_g_ rose for both the mica and MgO cases. Since all particle sizes and coherence lengths were smaller than the typical domain wall thickness (on the order of 100 nm [48]), an increase in particle size required a greater applied magnetic field to rotate the entire grain in its magnetization direction, resulting in a higher coercive force. For the CoFe/mica sample, the particle size increased from 17.4 nm at 300 °C to 42.2 nm at 400 °C. The coherence length also increased from 18 nm to 25.7 nm, an increase of about 42.8%. A similar trend was observed from 400 °C to 500 °C, where the particle size grew significantly from 42.2 nm to 108.2 nm, a 156.4% increase, resulting in an increase in H_c_ from 59.0 Oe to 232.5 Oe, as shown in Figure 4. However, in this temperature range, the correlation between particle size and H_c_ did not hold strictly. This could be due to an improved crystalline ordering, leading to a slight reduction in H_c_. For the CoFe/MgO sample, from 400 °C to 500 °C, the coherence length increased by only about 1 nm, while the particle size increased from 45.6 nm to 69.0 nm, a 51.3% increase, resulting in an increase in H_c_ from 93.1 Oe to 160.0 Oe. However, the particle size of CoFe on MgO increased from 34.1 nm at 300 °C to 45.6 nm at 400 °C, an increase of 11.5 nm, while the coherence length increased from 15.6 nm to 19.6 nm, about 25.6%. The dominant increase in coherence length led to a decrease in H_c_, which may also be attributed to improved crystalline ordering.

Furthermore, the surface morphology, H_c_, and squareness can be significantly affected by the applied strain. In the next section, the effects of the change of strain on mica are discussed.

### 3.3. Microscopic Strain on CoFe/Mica Under Bending Stress

**Figure 7:**
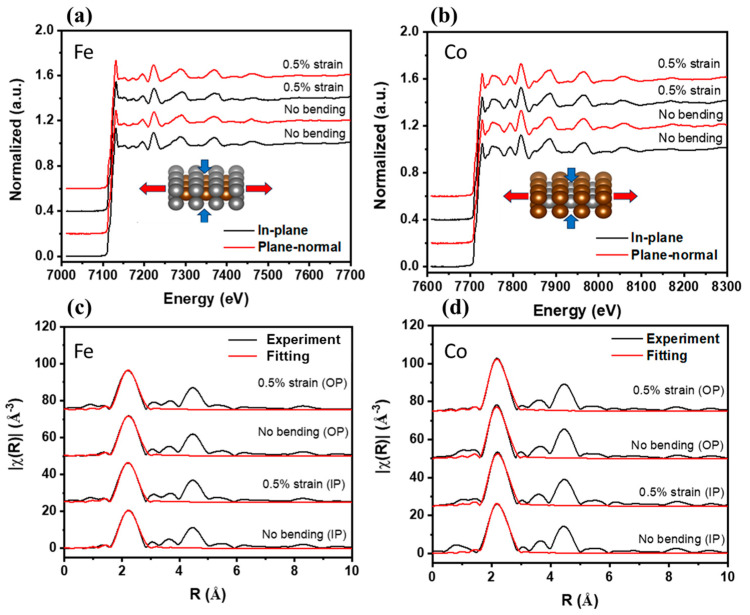
The normalized EXAFS measurements, including in-plane (IP) and plane-normal (OP) measurements under bending strain, are shown in (**a**,**b**). The inset arrows show tension and compression when strain is applied. The bond lengths obtained from R-space via Fourier transform are shown in (**c**,**d**), providing a detailed understanding of microscopic strain. The bond length data are summarized in Table 3.

During the bending process, the bending strain was calculated by Equation (2), which can be referenced from [49]. The detailed equation is shown as follows:

(2) $$S=\left(\frac{t_{f}+t_{s}}{2R}\right)\;\left(\frac{1+2\eta +\chi \eta^{2}}{\left(1+\eta \right)\left(1+\chi \eta \right)}\right)$$

where S represents the strain, and η = $t_{f}$/$t_{s}$, χ = $Y_{f}$/$Y_{S}$, and $t_{f}$, $t_{S}$ are taken as thickness of the deposited film and substrate, respectively. R is the radius of the bending curvature. $Y_{f}$ and $Y_{S}$ are Young’s modulus of thin film and substrate, respectively. The $Y_{f}$ of CoFe is about 207 GPa, obtained from [14], and the value of $Y_{s}$ for mica is about 190 GPa, taken from [50]. It can be calculated that, with R = 0.5 cm, the bending strain is 0.5%. However, this equation is valid only for the thin film with a thickness much larger than the coherence length, which is more than several micrometers. In the range of the sub-micrometer, the $Y_{f}$ usually is not accurate enough. Therefore, Equation (2) only provides us with a value of strain macroscopically; if the sample contains big cracks during the bending, this equation becomes invalid. To obtain the strain more accurately, we also applied XRD and EXAFS to understand the strain microscopically. Here, EXAFS with Fe and Co absorption K-edge under a bending strain of 0.5% macroscopically was chosen to compare macroscopic and microscopic strains. The change in microscopic strain obtained from the polarized EXAFS data can be found in the radial distribution in Figure 7. The change in bonding length from the fitting result can be separated into horizontal and vertical directions. With the applied strain, both Co-Co and Fe-Fe bond lengths increase in the horizontal direction. The microscopic strain of Co-Fe is −0.1% in the vertical direction. The results are summarized in Table 3. The difference between the applied 0.5% strain (macroscopic), that of 0.3% for the horizontal strain, and that of −0.1% for the vertical microscopic strain could be attributed to the 0.5% strain representing the macroscopic phenomenon, without considering the dislocations or micro-cracks of the thin film, which the EXAFS reveals in the microscopic phenomenon.

**Figure 8:**
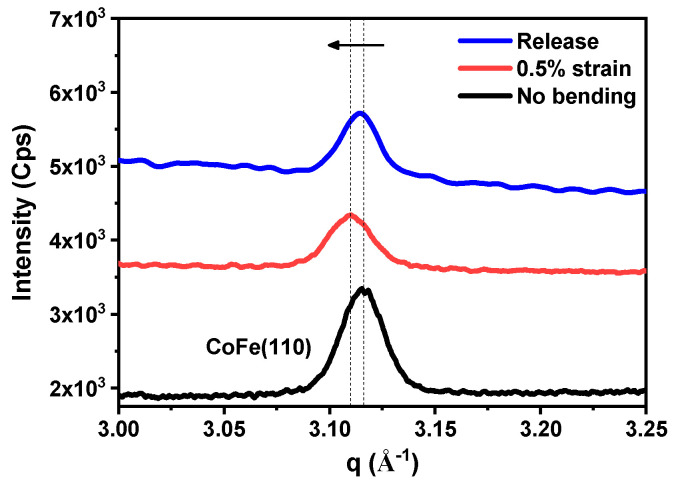
The in-plane d-spacing change of CoFe (110) measured by GIXRD before and after the 0.5% bending strain from Equation (2), the arrow indicated the peak shift of CoFe (110).

For a more detailed study of the microscopic phenomenon, the d-spacing change was also determined using the XRD experiment. Since the plane-normal scan overlapped with a signal coming from the mica substrate, GIXRD measurements were taken in the in-plane direction of CoFe. For the sample under an applied 0.5% strain, the results of d-spacings before and after the 0.5% strain are shown in Figure 8. The d-spacing was calculated using Bragg’s law and is summarized in Table 3. The microscopic in-plane strain was determined to be 0.25% from XRD, which is 50% smaller than the bending strain. However, after the applied strain was released, the d-spacing did not completely return to its original value. The calculated in-plane strain from XRD suggested partial strain relaxation. The microscopic strain data taken from XRD and EXAFS are the averaged value across the thickness of the thin film. To fully quantify the strain distribution and relaxation, further analysis is needed. A possible explanation could be the presence of dislocations and micro-cracks remaining on the crystal plane after the applied strain was released. XAS and XRD results revealed that the microscopic strain was smaller than the applied 0.5% strain, providing insights into both the macroscopic and microscopic behavior of the CoFe film under the applied strain bending process. On the other hand, CoFe films exhibited a magnetostrictive constant under a saturated magnetic field, reported as 70 ppm (about 0.007%) [51]. This value is 11 times smaller compared to the minimum bending strain without an applied magnetic field in this work, which was 0.08% (the value is calculated by Equation (2) with R = 3 cm). Therefore, the magnetostriction strain plays much less of a role in strain study here. In the meantime, the influence of H_c_ and squareness under bending strain is discussed later.

### 3.4. Bending Strain Effect on CoFe/Mica with Different Growth Temperatures

**Figure 9:**
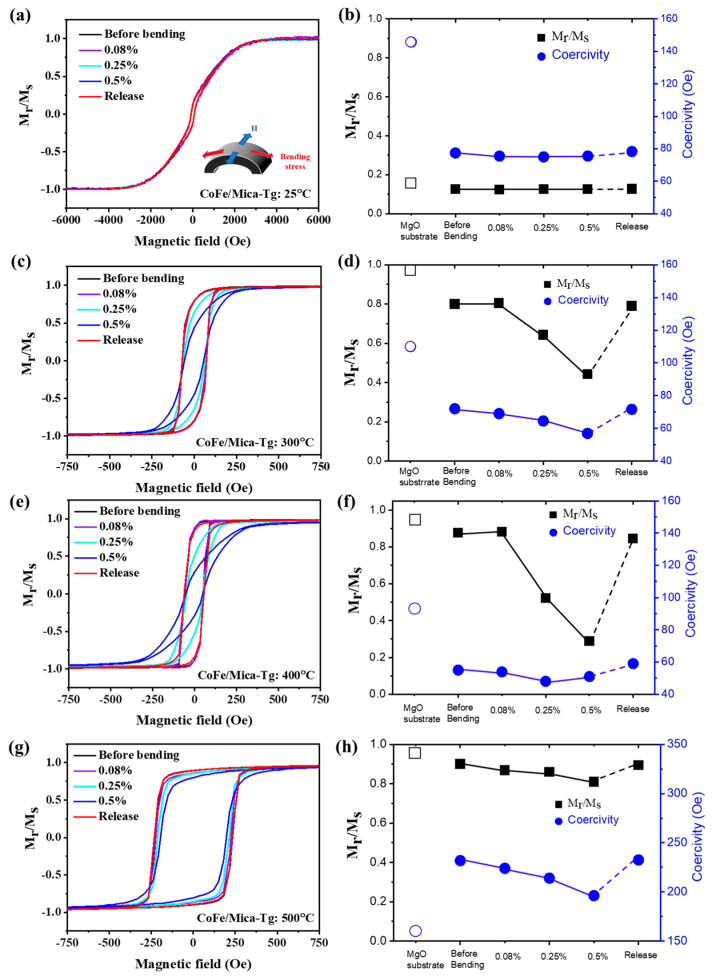
In-plane hysteresis loops of CoFe/mica were measured with different applied strains under the following growth temperatures: (**a**) 25 °C, (**c**) 300 °C, (**e**) 400 °C, and (**g**) 500 °C, respectively. The coercivity and squareness under different strains were concluded for (**b**) 25 °C, (**d**) 300 °C, (**f**) 400 °C, and (**h**) 500 °C, respectively.

The bending effect on H_c_ and squareness as a function of T_g_ can be observed in Figure 9a–h. Based on the experimental measurements, the squareness of T_g_ = 300 °C, 400 °C, and 500 °C was different. For the cases of 300 °C and 400 °C, the squareness decreased to approximately 0.5 at the 0.5% bending strain. On the other hand, the 500 °C case showed less squareness change compared to the cases of 300 °C and 400 °C, which was similar the case that was room temperature. At 25 °C, the magnetic moment was randomly oriented. Thus, the squareness did not change with applied strain. For 500 °C, due to the growth of the particle size, the dislocation density was higher, and the grain boundaries between each particle were larger than that of 300 °C and 400 °C. When strain was applied, part of the strain might be released by the dislocation slides and micro-cracks, reducing the effect of the applied strain. As a result, the squareness remained unchanged under strain, as observed at 300 °C and 400 °C. Moreover, the bending process also affected H_c_ under different T_g_ values. In Figure 9d,f,h, the bending process reveals that the bending strain could lower the coercivity in all T_g_ cases. These H_c_ and squareness values can be recovered after the bending is released. The only different case was that of T_g_ = 25 °C, which had a very poor squareness. From the crystalline study of CoFe on both mica and MgO substrates at 25 °C in Figure 5, neither of the CoFe films on MgO or mica exhibited long range ordering. The magnetic moment remained randomly oriented and not very aligned in the magnetization direction under the magnetic field. Thus, one thing that can be concluded is that both H_c_ and squareness are not sensitive to the applied strain on CoFe/mica at 25 °C, and the thin film magnetic sensor should not be prepared at room temperature by magnetic sputtering. The CoFe thin film grown at 400 °C, possessing the highest-order crystalline (see Figure 5a) with the highest AMR ratio, might be the best fit for a magnetic sensor. However, the poor squareness implies that the abruptness of magnetic sensors is not that sharp for some of the device’s applications.

### 3.5. Comparison of AMR Ratio with MgO Substrates and Mica Substrate Under Applied Strain

**Figure 10:**
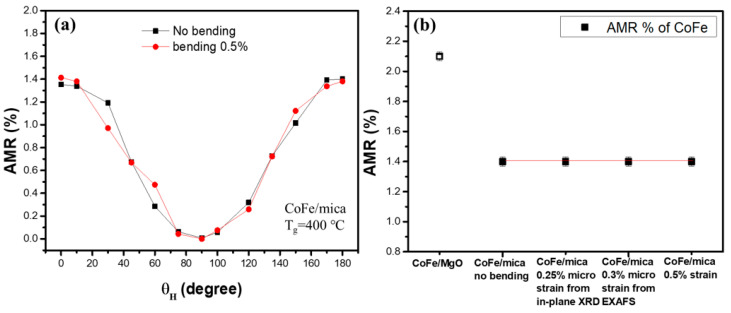
(**a**) AMR ratio before and after 0.5% strain effect; it is clear that the change in AMR ratio after the applied strain is small. (**b**) The comparison of the AMR ratio on the rigid MgO substrate and mica substrate before and after bending, including the cases of micro strain observed by EXAFS and in-plane XRD.

To verify whether the AMR ratio changes on mica and MgO under applied strain, the experimental AMR ratio under a bending state was collected and compared in Figure 10. In Figure 10a, the AMR ratios on mica substrate are almost the same under 0% and 0.5% bending strain [40]. Figure 10b shows the AMR ratio of CoFe films on mica under different strain conditions together with that of the rigid MgO substrate. In Figure 10, we can see that the in-plane AMR ratio is almost independent of strain in the bending mica substrate. This also indicated that the magnetostriction effect plays less of a role in the bending sample. This is a good property to possess if the AMR sensor is to be applied on the flexible wearable device.

## 4. Conclusions

In this work, we compared CoFe grown epitaxially on rigid MgO and flexible mica substrates and investigated their AMR ratios for potential magnetic sensor applications. Our findings indicate that a higher AMR ratio is observed on the MgO substrate compared to mica, which may be attributed to the lower lattice mismatch of CoFe on MgO. Additionally, the excellent epitaxial growth of CoFe on MgO results in a single domain structure, whereas on mica, CoFe exhibits partial epitaxy with three domains oriented at 120°, which might result in a lower AMR ratio. Furthermore, the AMR ratios observed in this epitaxial study are higher than those non-epitaxial films on polymer substrates. The different trends in the AMR ratio at θ_I_ = 0° and 45° observed on MgO and mica substrates may be due to the symmetric domain structure and the relative angles between θ_H_, θ_I_, and the easy axis. Finally, microscopic strain caused by misfit and bending strain on flexible mica, as determined by in-plane GIXRD and EXAFS, suggests the possibility that neither microscopic nor macroscopic strain significantly affect the AMR ratio on mica. This indicates that CoFe on flexible mica is a promising candidate for magnetic sensor applications. The results of this study facilitate the advancement of micro-scale magnetic sensor fabrication for the microelectronics industry.

## Figures and Tables

**Table 1:** Lattice mismatch of CoFe thin films grown on Mica and MgO substrates.

Lattice Mismatch	CoFe(100)/Mica(100)	CoFe(100)/MgO(110)
Previous reported data of mica [41] and MgO [42]	7.655%	−4.146%
This work is measured by plane-normal XRD	7.573%	−3.232%

**Table 2:** Comparison of particle size, coherence length, and magnetic property.

**CoFe/mica**					
T_g_	Particle size (nm)	Coherence length (nm)	Coercivity (Oe)	Squareness	Saturation magnetization (emu/cc)
300 °C	17.4	18.0	71.6	0.799	1562.9
400 °C	42.2	25.7	59.0	0.875	1653.3
500 °C	108.2	30.4	232.5	0.900	1701.3
**CoFe/MgO**					
T_g_	Particle size (nm)	Coherence length (nm)	Coercivity (Oe)	Squareness	Saturation magnetization (emu/cc)
300 °C	34.1	15.6	110.0	0.973	1476.6
400 °C	45.6	19.6	93.1	0.950	1383.9
500 °C	69.0	20.6	160.0	0.958	1474.9

**Table 3:** Microscopic strain from EXAFS and in-plane GIXRD.

**EXAFS Fitting Result**			
Experimental conditions	R_Fe-Co_ (Å)	R_Fe-Fe_ (Å)	Microscopic strain
No bending (in-plane)	2.439 ± 0.005	2.839 ± 0.009	0%
0.5% strain (in-plane)	2.444 ± 0.004	2.848 ± 0.008	0.3% (tensile)
No bending (plane-normal)	2.448 ± 0.005	2.839 ± 0.010	0%
0.5% strain (plane-normal)	2.449 ± 0.003	2.836 ± 0.006	−0.1%(compressive)
Calculate model from Artemi	2.467	2.849	
Experimental conditions	R_Co-Fe_ (Å)	R_Co-Co_ (Å)	Microscopic strain
No bending (in-plane)	2.446 ± 0.003	2.828 ± 0.006	0%
0.5% strain (in-plane)	2.449 ± 0.004	2.835 ± 0.009	0.3% (tensile)
No bending (plane-normal)	2.449 ± 0.004	2.830 ± 0.007	0%
0.5% strain (plane-normal)	2.450 ± 0.003	2.828 ± 0.005	−0.1%(compressive)
Calculate model from Artemis	2.467	2.849	
**In-plane XRD result**			
State	2theta (degree)	d-spacing (Å)	Microscopic strain
No bending	45.193° ± 0.005°	2.015 ± 0.001	0%
0.5% strain	45.105° ± 0.005°	2.020 ± 0.001	0.25%
Release	45.176° ± 0.005°	2.017 ± 0.001	0.09%

## Data Availability

The data presented in this study are available upon request from the corresponding author.
